# Correlation of intracranial and extracranial carotid atherosclerotic plaque characteristics with ischemic stroke recurrence: a high-resolution vessel wall imaging study

**DOI:** 10.3389/fneur.2024.1514711

**Published:** 2025-01-15

**Authors:** Shengyu Shao, Tianle Wang, Li Zhu, Yin Gao, Xian Fan, Yu Lu, Chengqun Qian, Manyu Zhang, Jinhua Qian

**Affiliations:** Department of Medical Imaging, The Second Affiliated Hospital of Nantong University, Nantong, China

**Keywords:** ischemic stroke, stroke recurrence, high-resolution vessel wall imaging, atherosclerosis, plaque characteristics

## Abstract

**Objectives:**

To evaluate the ability of the plaque characteristics of extracranial carotid and intracranial arteries to predict large atherosclerotic ischemic stroke recurrence via head and neck combined high-resolution vessel wall imaging (HR-VWI).

**Methods:**

This prospective cohort study included 169 patients with large atherosclerotic ischemic stroke who underwent head and neck combined HR-VWI from April 2022 to May 2023. The baseline clinical data and atherosclerotic plaque characteristics of the intracranial and extracranial carotid arteries were collected, and the patients were followed up for 1 year, with the endpoint event defined as recurrent ischemic stroke. Clinical and imaging data were compared between the recurrent and nonrecurrent groups. Independent risk factors associated with stroke recurrence were assessed via multivariate Cox regression analysis. The receiver operating characteristic (ROC) curves of the relevant variables were also plotted, and the area under the curve (AUC) was calculated to assess their ability to predict stroke recurrence. Kaplan–Meier survival curves were used to compare the probability of stroke recurrence.

**Results:**

During the 12-month follow-up, stroke recurrence occurred in 35 of the 169 patients. Multivariate Cox regression analysis revealed that the total number of intracranial and extracranial carotid plaques (*p* = 0.010) and coexisting extracranial carotid plaques and intracranial significantly enhanced plaques (*p* = 0.047) were independent risk factors for recurrent ischemic stroke. The AUCs for predicting stroke recurrence were 0.787 and 0.710, respectively. The Kaplan–Meier survival curve revealed that the risk of stroke recurrence was significantly greater in patients whose total number of intracranial and extracranial carotid plaques was >4.5 than in patients whose total number of plaques was <4.5 (*p* < 0.001) and was significantly greater in patients with coexisting extracranial carotid plaques and intracranial significantly enhanced plaques than in patients without coexisting plaques (*p* < 0.001).

**Conclusion:**

A greater total number of intracranial and extracranial carotid plaques and the coexistence of extracranial carotid plaques and intracranially significantly enhanced plaques are independent risk factors associated with recurrent ischemic stroke. Head and neck combined HR-VWI may provide new indicators for the prediction of stroke recurrence, thus helping clinicians identify high-risk patients and target therapy to reduce the recurrence of ischemic events.

## Introduction

Stroke is a common clinical cerebrovascular disease with high morbidity, disability, mortality, recurrence and economic burden and is the leading cause of death and disability among adults in China (1). Ischemic stroke is the main type of stroke, accounting for more than 80% of all strokes (2); it has an unstable prognosis and is prone to recurrence, especially in the early stages.

Atherosclerosis of the intracranial arteries and extracranial carotid arteries is the most common cause of ischemic stroke, and previous studies (3, 4) have demonstrated that many atherosclerotic plaque characteristics, including hyperintensity on T1-weighted imaging (T1WI), positive remodeling, plaque enhancement, and plaque burden, are correlated with the recurrence of ischemic stroke. However, atherosclerotic disease, as a systemic disease, usually affects multiple vascular beds. Therefore, simultaneous assessment of coexisting intracranial and extracranial atherosclerotic plaques may have greater predictive value for future cerebrovascular events than assessment of atherosclerotic plaques in a single vascular bed (5, 6).

Traditional vascular imaging techniques, such as computed tomography angiography (CTA), magnetic resonance angiography (MRA), and digital subtraction angiography (DSA), can only reveal the degree of stenosis and simple vessel wall information. Head and neck combined high-resolution vessel wall imaging (HR-VWI) can not only accurately assess the degree of stenosis but also has the unique advantages of providing potential pathological information within the vessel wall, visualizing plaque components, and accurately evaluating the morphology and signal characteristics of the arterial wall qualitatively and quantitatively. It is a repeatable and effective technique for the assessment of atherosclerotic disease (7).

This study aimed to evaluate the predictive value of extracranial carotid and intracranial plaque characteristics, as well as coexisting extracranial carotid and intracranial plaque characteristics, for large atherosclerotic ischemic stroke recurrence via head and neck combined HR-VWI and to identify the independent risk factors associated with stroke recurrence to provide guidance and assistance for the individualized treatment of patients with ischemic stroke.

## Materials and methods

### Patients

**Figure 1:**
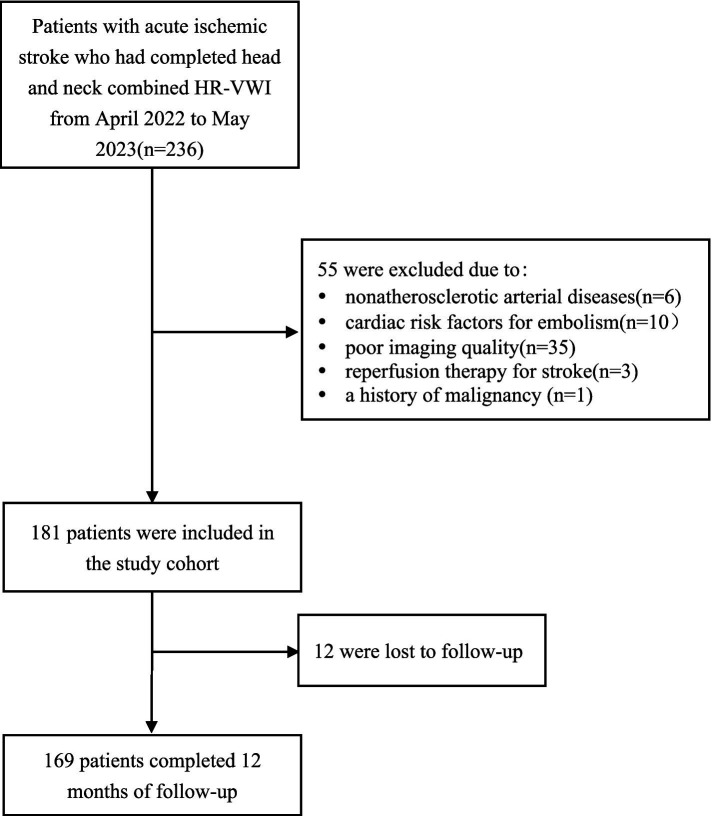
Flowchart showing the selection of patients.

This was a single-center prospective cohort study. Patients with acute ischemic stroke who underwent head and neck combined HR-VWI from April 2022 to May 2023 at the Second Affiliated Hospital of Nantong University were included in this study. The inclusion criteria were as follows: (1) age > 18 years; (2) acute ischemic stroke confirmed by DWI and clinically; (3) head and neck combined HR-VWI performed within 2 weeks of symptom onset; and (4) intracranial large atherosclerosis determined to be the etiology of the stroke by a multidisciplinary consultation. (5) Complete clinical information. The exclusion criteria were as follows: (1) nonatherosclerotic intracranial or extracranial arterial diseases, such as vasculitis, moyamoya disease, and dissection; (2) cardiac risk factors for embolism, such as rheumatic heart disease, myocardial infarction, and subacute bacterial endocarditis; (3) reperfusion therapy for stroke (intravenous thrombolysis/intravascular thrombus extraction/angioplasty/stenting); (4) angioplasty/stenting/eroticization during the follow-up period; (5) a history of malignancy or newly diagnosed malignancy at the time of follow-up; and (6) poor imaging quality. The patient selection flowchart is shown in Figure 1. This study followed the Declaration of Helsinki and was approved by the Ethics Committee of the Second Affiliated Hospital of Nantong University (2023KT208).

### Clinical data

Clinical data, including sex, age, history of hypertension, diabetes mellitus, smoking history, blood pressure, blood glucose, glycosylated hemoglobin, lipids, and renal function, were extracted from the clinical record system. Patients were also assessed for the National Institutes of Health Stroke Scale (NIHSS) score and Essen Stroke Risk Scale (ESRS) score.

### HR-VWI examination

A 3.0 T Siemens Magnetom Prisma MRI scanner (Siemens Healthcare, Erlangen, Germany) with a 64-channel combined head and neck coil was used. In addition to the routine DWI and TOF-MRA scanning sequences, 3D-T1-SPACE sequences were added before the contrast agent was injected and 10 min later. The subjects were kept in a supine position during image acquisition, with the head placed in the center of the coil, the head was fixed with an elastic cushion to avoid artifacts caused by head movement resulting in blurred images, and acoustic sponges were used in both ears to reduce noise hazards during scanning. The axial plane was positioned parallel to the anterior–posterior joint line, and the sagittal plane was positioned parallel to the midline of the brain. The scanning sequence and its parameters were as follows: TR 700 ms, TE 15 ms; FOV 220 mm × 220 mm, matrix 320 × 320, and voxel 0.3 mm × 0.3 mm × 0.7 mm. Gadopentetate dextran was used as the contrast agent at a dose of 0.1 mmol/kg.

### Image analysis

Image postprocessing was performed via a Siemens workstation. A second-year graduate neuroimaging-oriented student and a neuroradiologist with 5 years of experience analyzed the images. The intracranial arteries were evaluated mainly as the anterior cerebral artery A1–A2 segment, the middle cerebral artery M1–M2 segment, the posterior cerebral artery P1–P2 segment, the vertebral artery V4 segment, the basilar artery, and the internal carotid artery C3–C7 segment, and the extracranial carotid arteries were evaluated mainly as the common carotid artery end and the internal carotid artery C1–C2 segment. The location of atherosclerotic plaques was determined by 3D-T1-SPACE sequences combined with sagittal, coronal, and axial images, i.e., the presence of focal wall thickening with or without significant luminal stenosis. The location of the culprit plaque was also determined with reference to the intracranial vascular supply zone, which was the only lesion within the supplying artery in the area of the infarct focus or the most stenotic lesion in the presence of multiple plaques.

Intracranial culprit plaque characteristics were assessed on 3D-T1-SPACE images that optimally revealed the plaque by adjusting the images to be perpendicular to the long-axis level of the vessel, and the following indices were measured: outer wall area (OWA) at the plaque, lumen area (LA) at the plaque, OWA at the reference site, and wall area (WA) at the plaque = OWA-LA at the plaque. The degree of stenosis was measured on HR-VWI images with reference to the Warfain Apisin symptomatic intracranial arterial disease test (WASID) (8). The remodeling ratio (RR) = OWA at the plaque/OWA at the reference site, with positive remodeling occurring when the RR > 1.05 and negative remodeling occurring when the RR < 0.95 (9). Plaque burden = WA at the plaque/OWA at the plaque. The degree of plaque enhancement was determined by outlining the region of interest covering the whole plaque on 3D-T1-SPACE images before and after enhancement and recording the signal intensity (SI) before and after plaque enhancement. The plaque enhancement ratio = (SIpost-SIpre/SIpre), where SIpost is the SI of the enhanced plaque and where SIpre is the SI of the flat-scan plaque. Significant enhancement is defined when the SI of the enhanced plaque is equal to or greater than the enhancement signal of the pituitary stalk (10). Intraplaque hemorrhage (IPH) was defined as a high signal within the plaque on preenhancement 3D-T1-SPACE images that exceeded 150% of the adjacent muscle signal (11).

Extracranial carotid plaques are measured by the following indices: degree of stenosis, RR, IPH, and lipid-rich necrotic core (LRNC). The degree of stenosis was measured via the North American Symptomatic Carotid Endarterectomy Trial (NASCET) (12). LRNC is an iso-signal on TOF-MRA images and a high signal on 3D-T1-SPACE images and has no significant enhancement after enhancement (13).

Finally, extracranial carotid and intracranial atherosclerotic plaques were assessed simultaneously, the total number of intracranial and extracranial carotid plaques was calculated, and the presence of the following coexisting combinations was examined: (1) extracranial carotid plaques + intracranial plaques; (2) extracranial carotid plaques + intracranial stenosis (≥50%); (3) extracranial carotid plaques + intracranial positive remodeling; (4) extracranial carotid plaques + intracranial IPH; and (5) extracranial carotid plaques + intracranial significantly enhanced plaques.

We selected randomly 30 patients to test the intra-observer and inter-observer reproducibility of the quantitative and qualitative date. A 1-month interval was set to determine the intra-observer reproducibility to minimize memory bias.

### Follow-up and outcome assessment

Patients were followed up via face–to-face conversation or telephone at 3, 6, 9, and 12 months after discharge, with the endpoint event being recurrent ischemic stroke in the same vascular territory during the follow-up period. Recurrent stroke was defined as the worsening of neurological deficits or new neurological deficits and the presence of a new lesion in the relevant area of the brain, as confirmed by diffusion–weighted imaging (DWI) (14). The follow-up time was defined as the time of the endpoint event or, if no event occurred, the time of the most recent follow-up. Patients were categorized into recurrent and nonrecurrent groups on the basis of follow-up results.

### Statistical analysis

SPSS 26.0 software was used for statistical analysis. The normality of the quantitative data was tested via the Shapiro–Wilk test. Variables that conformed to a normal distribution are presented as the means ± standard deviations, and differences between groups were analyzed via two independent samples t tests. Information that was not normally distributed was described by medians and interquartile ranges, and differences between groups were analyzed via nonparametric tests. Count data are described as cases (%), and differences were analyzed via the chi-square test. Variables with *p* < 0.05 in the univariable Cox regression analysis were included in the multivariable Cox regression analysis to obtain independent characteristics associated with stroke recurrence. The receiver operating characteristic (ROC) curves of the relevant variables were also plotted, and the area under the curve (AUC) was calculated and compared via the Delong test. In addition, Kaplan–Meier survival curves and log-rank tests were used to compare the probability of recurrence after stroke for the variables of interest. The inter-observer and intra-observer reproducibility of quantitative date was evaluated by intraclass correlation coefficient (ICC) using a two-way random model with absolute agreement. The inter-observer and intra-observer reproducibility of qualitative date was evaluated by Cohen’s kappa value. A value of ICC and kappa≥0.75 indicates excellent agreement. *p* < 0.05 was considered a statistically significant difference.

## Results

### Baseline clinical data of patients

A total of 169 patients were included in this study, 35 of whom experienced stroke recurrence during an average follow-up time of 10.7 months. The median stroke recurrence time was 5 (2, 9.5) months. The median age of the patients in the recurrence group was 70 (65, 73) years, of which 62.9% were male, and the median age of the patients in the nonrecurrence group was 65 (56, 73) years, of which 61.9% were male. The age of the patients in the recurrence group was significantly greater than that of the patients in the nonrecurrence group (*p* = 0.024), and the remaining differences in the clinical data between the two groups were not statistically significant (*p* > 0.05), as shown in Table 1.

**Table 1:** Comparison of baseline clinical data between the recurrent and nonrecurrent groups.

Index	Recurrence group (*n* = 35)	Non-recurrence group (*n* = 134)	t/z/χ^2^	*p* value
Age, years	70(65, 73)	65(56, 73)	2.254	0.024
Male, n (%)	22(62.9%)	83(61.9%)	0.01	0.921
Systolic blood pressure, mmHg	146.37 ± 19.74	146.91 ± 20.60	−0.139	0.89
Diastolic blood pressure, mmHg	83(76, 90)	82.5(74, 91)	0.208	0.836
History of hypertension, n (%)	30(85.7%)	96(71.6%)	2.897	0.089
Diabetes mellitus, n (%)	14(40%)	46(34.3%)	0.39	0.532
Smoking history, n (%)	8(22.9%)	39(29.1%)	0.539	0.463
blood glucose, mmol/L	5.61(4.68, 6.99)	5.62(4.73, 7.40)	−0.671	0.502
Glycosylated hemoglobin, mmol/L	6.2(5.9, 7.6)	6.2(5.7,7.88)	0.132	0.895
Triglycerides, mmol/L	1.57(1.25, 1.88)	1.53(1.03, 1.98)	0.345	0.73
Total cholesterol, mmol/L	4.11(3.64, 4.49)	4.27(3.68, 4.95)	−0.793	0.428
HDL, mmol/L	1.03(0.92, 1.24)	1.11(0.97, 1.33)	−1.187	0.235
LDL, mmol/L	2.54(2.23, 2.86)	2.63(2.03, 3.14)	−0.202	0.840
Lipoprotein a, mmol/L	130(71, 290)	115(53.75, 252.75)	0.987	0.323
Urea nitrogen, mmol/L	5.20(4.04, 6.85)	5.37(4.28, 6.38)	−0.132	0.895
Creatinine, mmol/L	63(58.3, 70)	63.5(51.95, 72.55)	0.415	0.678
Uric acid, mmol/L	318(274, 387)	313.95(268.15, 363.95)	0.921	0.357
Hemoglobin,g/L	136.71 ± 17.28	138.38 ± 17.48	−0.503	0.615
NIHSS score	2(1, 4)	2(0, 4)	0.406	0.685
ESRS	2(2, 3)	2(1, 3)	1.420	0.156

HDL, high-density lipoprotein; LDL, low-density lipoprotein; NIHSS, National Institute of Health Stroke Scale; ESRS, Essen stroke risk score.

### Plaque characteristics of patients

**Figure 2:**
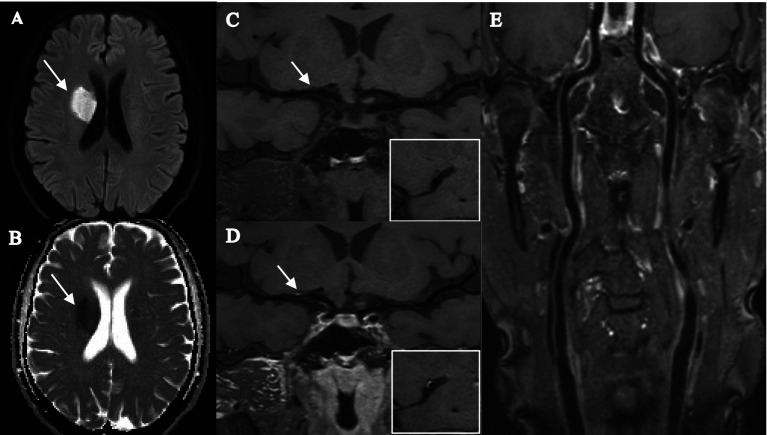
Female, 56 years old, was admitted to the hospital with orofacial tilt and left lower limb weakness for 2 days, and did not experience stroke recurrence during the 12-month follow-up. **(A,B)** DWI image shows a patchy hyperdense shadow in the right radiocoronal area with a corresponding reduced ADC value (arrow). **(C,D)** T1-SPACE images before and after enhancement show the presence of plaque in the M1 segment of the right middle cerebral artery (arrow), with low plaque burden, isosignal plaque before enhancement, mild enhancement after enhancement, and mild stenosis. **(E)** T1-SPACE image of the extracranial carotid artery does not show the presence of plaque.

**Figure 3:**
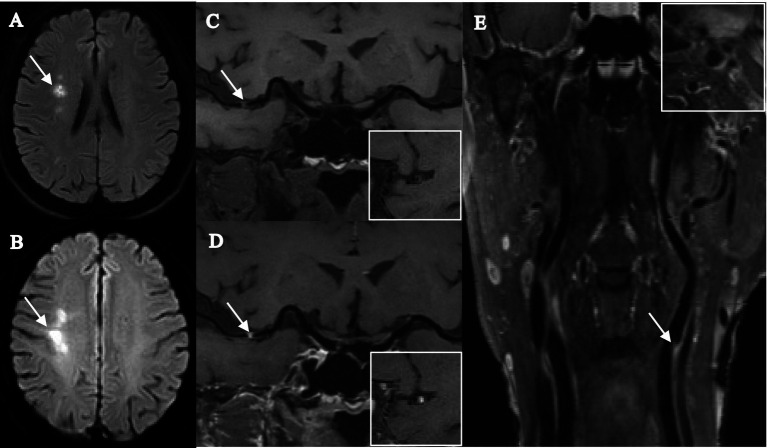
Female, 68 years old, was admitted to the hospital with numbness in the left limb, and stroke recurrence occurred at the 4-month follow-up. **(A)** DWI images before recurrence shows a patchy hyperdense shadow visible in the right corona radiata (arrow). **(B)** DWI images after recurrence shows a clumpy hyperdense shadow visible in the right centrum semiovale (arrow). **(C,D)** T1-SPACE images before and after enhancement show the presence of plaque in the M1 segment of the right middle cerebral artery (arrow), with high plaque burden, significant enhancement after enhancement, and severe stenosis. **(E)** T1-SPACE image of the extracranial carotid artery shows the presence of plaque at the beginning of the left internal carotid artery (arrow).

The intracranial and extracranial carotid plaque characteristics of the recurrent and nonrecurrent groups are shown in Table 2. Compared with the nonrecurrent group, the recurrent group had a greater total number of intracranial and extracranial carotid plaques and a greater incidence of positive remodeling of extracranial carotid arteries (*p* < 0.001), extracranial carotid plaques + intracranial plaques (*p* = 0.001), extracranial carotid plaques + intracranial stenosis (≥50%; *p* = 0.004), extracranial carotid plaques + intracranial IPH (*p* = 0.004), and extracranial carotid plaques + intracranial significantly enhanced plaques (*p* < 0.001). The results were statistically significant. The other characteristics were not significantly different between the two groups (*p* > 0.05). Typical imaging images of patients in the recurrent and nonrecurrent groups are shown in Figures 2, 3.

**Table 2:** Comparison of plaque characteristics between the recurrent and nonrecurrent groups.

Characteristic	Recurrence group (*n* = 35)	Non-recurrence group (*n* = 134)	t/z/χ^2^	*p* value
Total number of plaques	6(4, 7)	4(3, 5)	5.302	<0.001
Intracranial culprit plaque				
Degree of stenosis, %	55(30, 75)	40(26.75, 65)	1.205	0.228
Plaque burden	0.79 ± 0.099	0.76 ± 0.098	1.614	0.108
Remodeling ratio	1.10(0.89, 1.15)	1.06(0.92, 1.13)	0.921	0.357
Positive remodeling, n (%)	21(60%)	69(51.5%)	0.807	0.369
Plaque thickness, mm	1.7(1.4, 2.2)	1.6(1.4, 1.9)	1.738	0.082
Enhancement ratio	1.56(0.82, 1.76)	1.09(0.72, 1.51)	1.755	0.079
IPH, n (%)	11(31.4%)	40(29.9%)	0.033	0.856
Significant enhancement, n (%)	20(57.1%)	58(43.3%)	2.145	0.143
Extracranial carotid plaque
Stenosis (≥50%), n (%)	4(11.4%)	3(2.2%)	3.815	0.051
Positive remodeling, n (%)	19(54.3%)	32(23.9%)	12.176	<0.001
LRNC, n (%)	3(8.6%)	12(9%)	0.005	0.943
IPH, n (%)	2(5.7%)	3(2.2%)	0.271	0.603
Coexisting atherosclerotic plaques				
Extracranial carotid plaques + intracranial plaques, n (%)	23(65.7%)	45(33.6%)	11.915	0.001
Extracranial carotid plaques + intracranial stenosis (≥50%), n (%)	14(40%)	23(17.2%)	8.463	0.004
Extracranial carotid plaques + intracranial positive remodeling, n (%)	12(34.3%)	29(21.6%)	2.415	0.120
Extracranial carotid plaques + intracranial IPH, n (%)	12(34.3%)	18(13.4%)	8.265	0.004
Extracranial carotid plaques + intracranial significantly enhanced plaques, n (%)	21(60%)	24(17.9%)	25.164	<0.001

IPH, Intraplaque hemorrhage; LRNC, lipid-rich necrotic core.

The kappa values were, respectively, 1.00, 0.95, 0.90, 0.89, 1.00, 0.98 and 0.93 for the intra-observer reproducibility in the identification of intracranial IPH, intracranial significantly enhanced plaques, intracranial positive remodeling, extracranial carotid stenosis (≥50%), extracranial carotid IPH, extracranial carotid LRNC and extracranial carotid positive remodeling. For the inter-observer reproducibility, the values of the same parameters were 0.98, 0.92, 0.88, 0.85, 0.99, 0.93 and 0.90, respectively. The intra-observer ICC values of plaque thickness, plaque burden, degree of stenosis and remodeling ratio of intracranial culprit plaques were 0.95 (95% CI 0.90–0.98), 0.89 (95% CI 0.75–0.94), 0.87 (95% CI 0.73–0.95) and 0.80 (95% CI 0.62–0.89), respectively. The inter-observer ICC values for the same parameters were as follows: 0.96 (95% CI 0.92–0.99), 0.94 (95% CI 0.85–0.97), 0.85 (95% CI 0.72–0.91) and 0.84 (95% CI 0.72–0.91).

### Independent risk factor analysis for stroke recurrence

Univariable and multivariable Cox regression analyses were used to detect risk factors associated with stroke recurrence and variables with *p* < 0.05 in the univariable analysis were included in the multivariable Cox regression model. The results revealed that the total number of intracranial and extracranial carotid plaques (HR = 1.327; 95% CI 1.070–1.647; *p* = 0.010) and coexisting extracranial carotid plaques and intracranial significantly enhanced plaques (HR = 5.375; 95% CI 1.025–28.171; *p* = 0.047) were independent risk factors for stroke recurrence, as shown in Tables 3, 4.

**Table 3:** Univariable cox regression of the significant indicators associated with stroke recurrence.

Risk factors	HR(95%CI)	*p* value
Age	1.043(1.007,1.080)	0.018
Total number of intracranial and extracranial carotid plaques	1.568(1.334,1.844)	<0.001
Positive remodeling of extracranial carotid arteries	3.052(1.569,5.938)	0.001
Extracranial carotid plaques + intracranial plaques	3.171(1.577,6.376)	0.001
Extracranial carotid plaques + intracranial stenosis (≥50%)	2.755(1.400,5.422)	0.003
Extracranial carotid plaques + intracranial IPH	2.903(1.443,5.837)	0.003
Extracranial carotid plaque + intracranial significantly enhanced plaques	5.094(2.586,10.034)	<0.001

IPH, Intraplaque hemorrhage; HR hazard ratio; CI confidence interval.

**Table 4:** Multivariate cox regression of the significant indicators associated with stroke recurrence.

Risk factors	HR(95%CI)	*p* value
Age	1.032(0.990,1.075)	0.136
Total number of intracranial and extracranial carotid plaques	1.327(1.070,1.647)	0.010
Positive remodeling of extracranial carotid arteries	1.326(0.433,4.062)	0.621
Extracranial carotid plaques + intracranial plaques	0.248(0.038,1.604)	0.143
Extracranial carotid plaques + intracranial stenosis (≥50%)	1.809(0.727,4.503)	0.203
Extracranial carotid plaques + intracranial IPH	0.822(0.323,2.089)	0.680
Extracranial carotid plaque + intracranial significantly enhanced plaques	5.375(1.025,28.171)	0.047

IPH, Intraplaque hemorrhage; HR hazard ratio; CI confidence interval.

**Figure 4:**
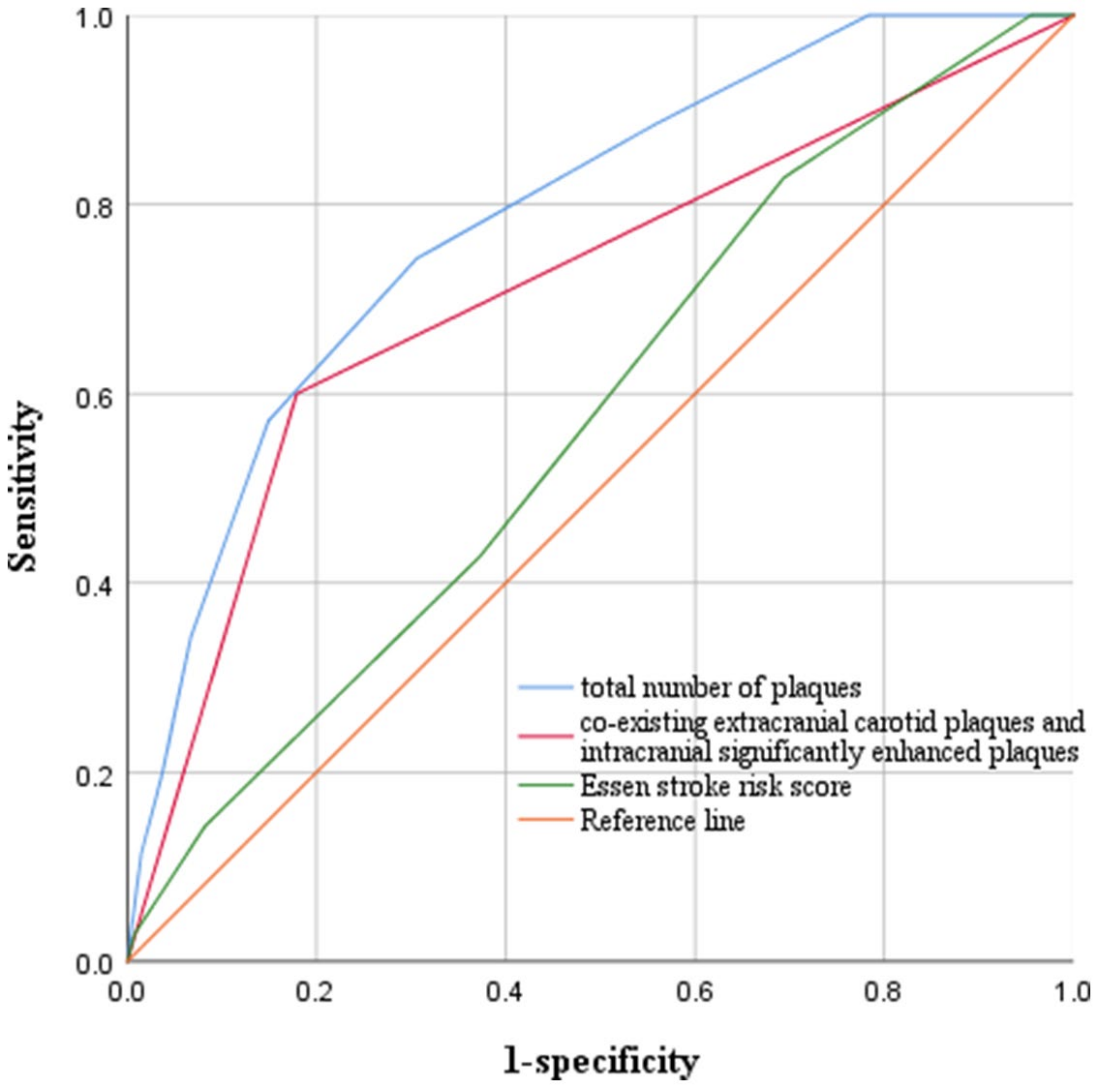
ROC curves for predicting stroke recurrence. The optimal cutoff value of the total number of plaques to predict stroke recurrence was 4.5, with an AUC value of 0.787, a sensitivity of 74.3%, and a specificity of 69.4%. The AUC value of coexisting extracranial carotid plaques and intracranial significantly enhanced plaques to predict stroke recurrence was 0.710, with a sensitivity of 60% and a specificity of 82.1%. The AUC value for ESRS was 0.575.

We plotted ROC curves for coexisting extracranial carotid plaques and intracranial significantly enhanced plaques, the total number of intracranial and extracranial carotid plaques, and ESRS to predict stroke recurrence (Figure 4). On the basis of the ROC curve analysis, the optimal cutoff value of the total number of plaques to predict stroke recurrence was 4.5, with an AUC value of 0.787 (95% CI 0.705–0.869), a sensitivity of 74.3%, and a specificity of 69.4%. The AUC value of coexisting extracranial carotid plaques and intracranial significantly enhanced plaques to predict stroke recurrence was 0.710 (95% CI 0.607–0.814), with a sensitivity of 60% and specificity of 82.1%. The AUC value for the ESRS was 0.575 (95% CI 0.473–0.677). The Delong test revealed a statistically significant difference between the AUC values of coexisting extracranial carotid plaques and intracranially significantly enhanced plaques and the ESRS (*z* = 2.399, *p* = 0.016) and between the AUC values of the total number of plaques and the ESRS (*z* = 3.943, *p* < 0.001).

### Kaplan–Meier survival curve analysis

**Figure 5:**
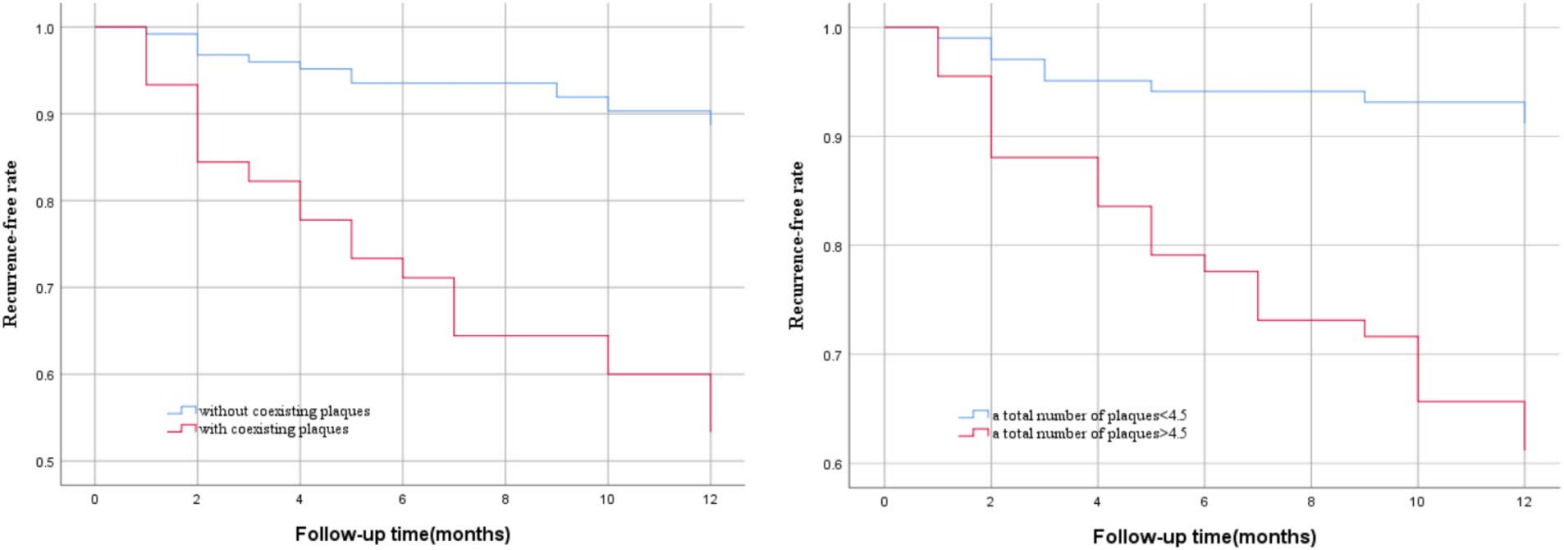
Kaplan–Meier survival curves of stroke recurrence probability. Patients with a total number of plaques> 4.5 (*p* < 0.001) and coexisting extracranial carotid plaques and intracranial significantly enhanced plaques (*p* < 0.001) had a higher risk of stroke recurrence during follow-up.

We used Kaplan–Meier survival curve analysis to determine the risk of stroke recurrence according to the optimal cutoff value for the total number of intracranial and extracranial carotid plaques (4.5) and the coexistence of extracranial carotid plaques and significantly enhanced intracranial plaques. The vertical axis represents the rate of no recurrence and the horizontal axis represents the follow-up time. By log-rank tests we found that the risk of stroke recurrence was significantly greater in patients with a total number of plaques>4.5 than in patients with a total number of plaques<4.5 (*p* < 0.001), and the risk of stroke recurrence was significantly greater in patients with coexisting extracranial carotid plaques and intracranially significantly enhanced plaques than in patients without coexisting plaques (*p* < 0.001), as shown in Figure 5.

## Discussion

In this prospective cohort study, we found that a greater total number of intracranial and extracranial carotid plaques and the coexistence of extracranial carotid plaques and intracranially significantly enhanced plaques were independent risk factors associated with ischemic stroke recurrence. Both had good predictive ability for stroke recurrence, whereas ESRS did not.

Atherosclerosis is a systemic disease, and no independent risk factors associated with stroke recurrence were found when extracranial carotid and intracranial atherosclerotic plaque characteristics were analyzed separately in this study, whereas coexisting plaques and the total number of plaques in the intracranial and extracranial carotid arteries were significantly associated with stroke recurrence, suggesting that simultaneous assessment of multiple vascular beds is more valuable than assessment of a single vascular bed in the prediction of stroke recurrence. In line with previous studies, Suo et al. (15) analyzed the relationship between intracranial and extracranial large atherosclerosis and prognosis through multivariate Cox regression by enrolling 806 patients with less severe acute stroke and finally reported that the coexistence of atherosclerosis with >50% stenosis in the intracranial and extracranial arteries can result in a significant increase in the rate of stroke recurrence by a factor of approximately 3.4. Another study (16) also found that patients with symptomatic vascular disease in multiple arterial beds had a poor long-term prognosis for stroke or TIA, and that the prognosis worsened with the number of vessels involved. This may be due to the increased risk of plaque rupture due to more plaques and the involvement of more vascular beds, further increasing the probability of stroke recurrence. Moreover, the number of atherosclerotic risk factors increases with the number of affected vascular beds, including lipids and smoking, which are independently associated with stroke recurrence and are important targets for secondary prevention of stroke (17). Thus, the number of affected vascular beds may be able to be used as a summative measure to predict stroke recurrence, which further demonstrates the additive value of multivessel bed assessment for stroke recurrence prediction. Clinicians can try to include assessment of the carotid artery to further predict the prognosis of stroke patients.

However, owing to the limitations of the examination technique, most of the previous studies were limited to the coexistence of intracranial and extracranial atherosclerosis and the degree of stenosis and did not involve other characteristics of the plaques. In this study, head and neck combined HR-VWI was employed to perform the examination. Magnetic resonance can provide high spatial resolution and excellent soft tissue contrast, which can not only identify atherosclerotic plaques with mild to moderate stenosis more accurately but also visualize and characterize the vessel wall and reveal more pathological characteristics of plaques (18) to further analyze the relationship between the coexistence of high-risk plaque characteristics and stroke recurrence. A similar study was previously performed by Wu et al. (19). They followed up 97 stroke patients who underwent head and neck combined HR-VWI, and 21 patients experienced stroke recurrence. Multivariate Cox regression analysis revealed that the coexistence of intracranial T1WI high-signal plaques and extracranial carotid atherosclerotic plaques was an independent risk factor for stroke recurrence, with greater predictive value than the assessment of extracranial carotid atherosclerosis alone. Jiang et al. (20) also reported that the coexistence of severe stenosis of intracranial arteries with greater plaque burden, intraplaque calcification, and LRNC in extracranial carotid arteries was independently associated with ipsilateral acute cerebrovascular events through magnetic resonance vessel wall imaging. Although both of these results differ from the final results of the present study, the coexistence of plaque characteristics found by HR-VWI is more valuable for the prediction of stroke recurrence than the coexistence of atherosclerosis alone is, and this could be further explored in the future.

As one of the important characteristics of plaque vulnerability, significant plaque enhancement has been demonstrated in many previous studies to be correlated with ischemic stroke recurrence (21, 22). In this study, although we did not observe a strong relationship between significant enhancement of intracranial plaques and stroke recurrence, the coexistence of extracranial carotid plaques and intracranial significantly enhanced plaques was independently associated with stroke recurrence, indicating that significant plaque enhancement can still significantly improve the ability to predict recurrence. This may be because significant plaque enhancement generally represents plaque inflammation, macrophage infiltration and neovascularization, and the accumulation of inflammatory cells in specific areas of the plaque can further lead to plaque hemorrhage and rupture, which increases the instability of the plaque and consequently leads to the occurrence of stroke (23). However, a sufficiently reliable pathological basis for the correlation between plaque enhancement and stroke recurrence is still lacking, and larger prospective studies are needed for further validation.

In addition, the degree of arterial stenosis is one of the traditional predictors of stroke recurrence, and a study by Li et al. (24) reported that the stenosis rate of the responsible vessel was significantly greater in patients with recurrent ischemic stroke than in patients without recurrence, which can be used as an independent risk factor for predicting stroke recurrence. However, our study did not find a significant association between moderate-to-severe stenosis of extracranial carotid and intracranial arteries and stroke recurrence, and the fact that the majority of plaques showed positive remodeling may be one of the main reasons. Plaques with positive remodeling are considered to have a greater risk of rupture and are more likely to lead to the occurrence and recurrence of stroke (25), whereas plaques with positive remodeling often show mild stenosis; thus, there is a limitation in judging the vulnerability of plaques by the degree of stenosis, neglecting the effect of plaque remodeling. Plaque burden and IPH are also important characteristics indicating plaque vulnerability (26), and many previous studies (27, 28) have shown that both are significantly associated with ischemic stroke recurrence and are important predictors of stroke recurrence. However, this is contrary to the results of our study, which did not find a significant association between a larger plaque burden and the presence of IPH with stroke recurrence, which may be due to the low occurrence of both in all plaques and the fact that our study was a single-center, smaller-sample-size trial, which led to the masking of the association between both and stroke recurrence. Thus, more multicenter trials with large sample sizes are needed to explore this topic further in the future.

The study also revealed that the ESRS score was not a good predictor of stroke recurrence, with an AUC of only 0.575, and a retrospective cohort study by Lv et al. (29) also revealed that the AUC of the ESRS score for the prediction of stroke recurrence was 0.595, which was similar to our study, indicating that the indicators included in the score lacked good predictive ability for stroke recurrence. In contrast, the AUCs for the total number of intracranial and extracranial carotid plaques and the coexistence of extracranial carotid plaques and intracranially significantly enhanced plaques were significantly greater than those of the ESRS, suggesting that the clinic may try to apply these two indicators to assess the risk of stroke recurrence, in addition to traditional assessment tools.

Our study also has the following limitations. First, this is a single-center study with a small sample size, which may obscure the association between certain low-incidence plaque characteristics and stroke recurrence, and further multicenter studies with large sample sizes are needed in the future. Second, atherosclerosis is a systemic disease, and only intracranial arteries and extracranial carotid arteries were evaluated in the present study. Research on more vessels should be included in the future. Third, in addition to plaque characteristics, hemodynamics and collateral circulation formation are strongly associated with stroke recurrence and should be included in future studies. Finally, atherosclerosis is a dynamic process, and HR-VWI was not performed during the follow-up period in this study.

## Conclusion

In conclusion, a greater total number of intracranial and extracranial carotid plaques and the coexistence of extracranial carotid plaques and significantly enhanced intracranial plaques are independent risk factors associated with ischemic stroke recurrence. Head and neck combined HR-VWI may provide new indicators for the prediction of stroke recurrence, thus helping clinicians identify high-risk patients and target therapy to reduce the recurrence of ischemic events.

## Data Availability

The raw data supporting the conclusions of this article will be made available by the authors, without undue reservation.
